# Neurons derived from sporadic Alzheimer’s disease iPSCs reveal elevated TAU hyperphosphorylation, increased amyloid levels, and GSK3B activation

**DOI:** 10.1186/s13195-017-0317-z

**Published:** 2017-12-01

**Authors:** Anna Ochalek, Balázs Mihalik, Hasan X. Avci, Abinaya Chandrasekaran, Annamária Téglási, István Bock, Maria Lo Giudice, Zsuzsanna Táncos, Kinga Molnár, Lajos László, Jørgen E. Nielsen, Bjørn Holst, Kristine Freude, Poul Hyttel, Julianna Kobolák, András Dinnyés

**Affiliations:** 1 Molecular Animal Biotechnology Laboratory, Szent István University, H-2100 Gödöllő, Hungary; 2 BioTalentum Ltd., Aulich Lajos Street 26, H-2100 Gödöllő, Hungary; 3 Department of Anatomy, Embryology and Histology, Faculty of Medicine, University of Szeged, H-6700 Szeged, Hungary; 4 Department of Anatomy, Cell and Developmental Biology, Eötvös Loránd University, H-1117 Budapest, Hungary; 5 Neurogenetics Clinic & Research Laboratory, Danish Dementia Research Centre, Department of Neurology, Rigshospitalet, University of Copenhagen, Copenhagen, Denmark; 6 Bioneer A/S, 2970 Hørsholm, Denmark; 7 Department of Veterinary and Animal Sciences, University of Copenhagen, 1870 Copenhagen, Denmark; 8 Present address: University Department of Otolaryngology, Head and Neck Surgery, University of Tübingen, 72076 Tübingen, Germany

**Keywords:** Familial Alzheimer’s disease, Sporadic Alzheimer’s disease, Induced pluripotent stem cells, Amyloid β, TAU pathology, Hyperphosphorylation, GSK3B

## Abstract

**Background:**

Alzheimer’s disease (AD) is the most common type of dementia, affecting one in eight adults over 65 years of age. The majority of AD cases are sporadic, with unknown etiology, and only 5% of all patients with AD present the familial monogenic form of the disease. In the present study, our aim was to establish an in vitro cell model based on patient-specific human neurons to study the pathomechanism of sporadic AD.

**Methods:**

We compared neurons derived from induced pluripotent stem cell (iPSC) lines of patients with early-onset familial Alzheimer’s disease (fAD), all caused by mutations in the *PSEN1* gene; patients with late-onset sporadic Alzheimer’s disease (sAD); and three control individuals without dementia. The iPSC lines were differentiated toward mature cortical neurons, and AD pathological hallmarks were analyzed by RT-qPCR, enzyme-linked immunosorbent assay, and Western blotting methods.

**Results:**

Neurons from patients with fAD and patients with sAD showed increased phosphorylation of TAU protein at all investigated phosphorylation sites. Relative to the control neurons, neurons derived from patients with fAD and patients with sAD exhibited higher levels of extracellular amyloid-β 1–40 (Aβ_1–40_) and amyloid-β 1–42 (Aβ_1–42_). However, significantly increased Aβ_1–42_/Aβ_1–40_ ratios, which is one of the pathological markers of fAD, were observed only in samples of patients with fAD. Additionally, we detected increased levels of active glycogen synthase kinase 3 β, a physiological kinase of TAU, in neurons derived from AD iPSCs, as well as significant upregulation of amyloid precursor protein (APP) synthesis and APP carboxy-terminal fragment cleavage. Moreover, elevated sensitivity to oxidative stress, as induced by amyloid oligomers or peroxide, was detected in both fAD- and sAD-derived neurons.

**Conclusions:**

On the basis of the experiments we performed, we can conclude there is no evident difference except secreted Aβ_1–40_ levels in phenotype between fAD and sAD samples. To our knowledge, this is the first study in which the hyperphosphorylation of TAU protein has been compared in fAD and sAD iPSC-derived neurons. Our findings demonstrate that iPSC technology is suitable to model both fAD and sAD and may provide a platform for developing new treatment strategies for these conditions.

**Electronic supplementary material:**

The online version of this article (doi:10.1186/s13195-017-0317-z) contains supplementary material, which is available to authorized users.

## Background

There are nearly 47 million people living with dementia worldwide, which is predicted to double every 20 years, increasing to more than 131 million by 2050. Alzheimer’s disease (AD) is the best characterized among them, and it accounts for 50–60% of all dementia cases [1]. This common neurodegenerative disease is clinically characterized by a progressive and gradual cognitive impairment, synapse loss, and substantial loss of neurons in later stages. Owing to the disease’s heterogeneity, the etiology of AD is still not very well understood. Most cases of early-onset AD are linked to autosomal dominant inherited mutations in the genes encoding amyloid precursor protein (*APP*), presenilin 1 (*PSEN1*), and presenilin 2 (*PSEN2*). These cases are referred to as familial Alzheimer’s disease (fAD) and are well characterized. In contrast, the etiology of the remaining 95% cases of late-onset AD, often referred to as sporadic Alzheimer’s disease (sAD), requires further investigation owing to the various factors involved in the pathology, including genetic and environmental exposures [2]. Moreover, cellular changes in the brain precede the first clinical symptoms by 10–15 years, and there is a lack of early diagnostic biomarkers for the prodromal stages of AD. Currently, there is no cure for AD, and the available medications can only slow down the progression of dementia and slightly improve the quality of life of the patients [3].

Two well-defined pathological hallmarks of AD have been described: the formation of extracellular amyloid plaques and the development of intracellular neurofibrillary tangles (NFTs) formed by aggregated hyperphosphorylated TAU protein [4]. The amyloid plaques are composed mainly of Aβ and form aggregates of regularly ordered amyloid fibers [5, 6]. Aβ peptides are derived from the sequential cleavage of APP by the proteolytic enzymes β- and γ-secretase. The cleavage of APP by β-secretase results in production of a large, secreted, derivative soluble amyloid precursor protein β protein and a 99-amino acid membrane-associated fragment (CT99) [7]. The γ-secretase complex cleaves within the transmembrane fragment of CT99 at various positions and generates peptides with differing lengths of the C-terminus (Aβ_1–37_, Aβ_1–38_, Aβ_1–40_, Aβ_1–42_, Aβ_1–43_; reviewed in [8]). Among these, Aβ_1–40_ is the most abundant isoform (~80–90% of the total Aβ peptide) [9], whereas the Aβ_1–42_ variant is the main isoform of the amyloid deposits found in AD [10, 11]. Aβ_1–42_ peptides have higher self-aggregating properties than the less hydrophobic, smaller Aβ species, and they have the ability to form oligomers. These diffusible, nonfibrillar, soluble, low-molecular-weight oligomers have neurotoxic effects [9]. It has been shown that soluble Aβ oligomers disrupt axonal transport [12] and cause synapse deterioration, reversal of spines, reduction of long-term potentiation (LTP), and induction of long-term depression (LTD), as well as altered mitochondrial distribution and dynamics in neurons [13]. Inhibition of LTP [14] and reversal of LTD [15] observed in the rat dentate gyrus are associated with impairment of cognitive function. Binding of Aβ oligomers to specific binding sites on neurons (reviewed in [16]) leads to redistribution of synaptic proteins and activation of ionotropic glutamate receptors [17]. Aβ oligomers inhibit LTP by increasing *N*-methyl-d-aspartate (NMDA) response through glutamate ionotropic receptor NMDA type subunit 2B. Furthermore, these molecules may cause a rapid depolarization of neurons and release of glutamate due to Ca^2+^ entry, leading to excitotoxicity and synapse loss [17].

Additionally, mutations in *PSEN1*, *PSEN2*, and *APP* cause imbalances between synthesis and degradation of Aβ. For instance, *PSEN* mutations lead to a partial loss of function in the γ-secretase complex and consequently to incomplete Aβ digestion [18], whereas most of the mutations in *APP* have been found to increase production of Aβ peptides [19]. The above-described gene aberrations result in many abnormal cellular responses, such as mitochondrial dysfunction, inflammation, activation of microglia, and neuronal loss [20].

The main mechanism in amyloid pathology in fAD is the increased production of Aβ species, whereas decreased Aβ clearance is postulated in sAD, which is modulated by the apolipoprotein E (*APOE*) genotype. APOE isoforms differentially regulate Aβ aggregation and clearance in the brain and have distinct functions in regulating brain lipid transport, glucose metabolism, neuronal signaling, neuroinflammation, and mitochondrial function (reviewed in [21]). There are at least three alleles of the *APOE* gene—ε2, ε3, and ε4—coding different isoforms of the protein. The most common allele (~60% in the population) is ε3. Individuals carrying the ε4 allele are at increased risk of AD compared with those carrying the ε3 allele, whereas the ε2 allele is known to decrease the risk.

The second characteristic of AD is TAU-based neurofibrillary pathology. TAU is a microtubule-associated protein (MAPT) required for stabilizing microtubules, the major component of the neuronal cytoskeleton. This protein is involved in neurite outgrowth, maintenance of neuronal polarity, and axonal transport (reviewed in [22]). Phosphorylation of TAU is regulated by kinases and phosphatases, and their actions on TAU molecules are required for proper neuronal growth. Site-specific phosphorylation of TAU plays a crucial role in microtubule stabilization, dynamic behavior, and spatial organization of microtubules in neurons and axonal transport regulation (reviewed in [23]).

In AD, hyperphosphorylated TAU (AD pTAU) spontaneously aggregates into paired helical filaments (PHFs) and form NFTs. Abnormal phosphorylation of TAU impairs microtubule-binding capacity and leads to microtubule destabilization [24]. These disturbances may cause improper anterograde and retrograde axonal transport and perturb intraneuronal signaling, including synaptic transmission (reviewed in [25]).

TAU dysfunction is also related to other forms of dementia, including frontotemporal lobar degeneration, commonly referred as *frontotemporal dementia* (FTD), which affects people before the age of 65 [26]. Mutations in several different genes can cause FTD; however, the granulin precursor (*GRN*) and *MAPT* genes are the most frequent harborers of such mutations. This genetic alteration induces TAU hyperphosphorylation and aberrant NFT formation [27, 28].

Studies on normal human brain tissue have revealed phosphorylation of TAU at several serine and threonine residues, whereas in AD more than 40 different phosphorylation sites have been identified [29]. Most of the sensitive TAU hyperphosphorylation sites are located at the microtubule-binding repeat domain in the proline-rich regions [30]. These regions include serine and threonine residues in serine-proline and threonine-proline motifs, and therefore they are targets of proline-directed protein kinases such as glycogen synthase kinase 3β (GSK3B) [31]. Studies have clearly demonstrated that phosphorylation of TAU by GSK3B and cyclin-dependent kinase 5 reduces the affinity of TAU to bind to microtubules [31], whereas phosphorylation of the serines within the KXGS motifs destructively affects TAU-microtubule interactions [32]. Recent data have shown that Aβ accumulated in the AD brain can activate kinases that promote TAU phosphorylation, including GSK3B [33]. GSK3B phosphorylates serine and threonine residues in TAU PHFs, and its activity corresponds with increased Aβ expression and Aβ-mediated neuronal death. Additionally, active forms of GSK3B were observed in cell lines with presenilin mutations [34].

Current understanding of AD pathogenesis is limited owing to the difficulties in obtaining and culturing human brain tissue and live neurons and inability to model the disease. The recently developed induced pluripotent stem cell (iPSC) technology may provide a new platform to create reliable human disease models for better understanding the pathological mechanisms of AD and for establishment new therapeutic strategies. An increasing number of studies have shown that neurons derived from patient-specific iPSCs can recapitulate the major cellular phenotypes of AD and hence may offer themselves for disease modeling or drug development [35]. In regard to AD, a few research groups have reported elevated levels of Aβ_1–42_ and increased Aβ_1–42_/Aβ_1–40_ ratio in neurons differentiated from iPSCs of patients with fAD [36–39], whereas authors of one report detected an increased secretion of Aβ_1–42_ in only one patient with sAD [40]. Additionally, AD neurons revealed elevated phosphorylation of TAU at Thr231 as well as an increased level of active GSK3B, and they accumulated large RAB5A-positive early endosomes [40].

We report the generation and neural differentiation of iPSCs derived from several patients with fAD and sAD, as well as from control individuals without dementia. The aim of our present study was to establish an in vitro cellular model that reveals major phenotypes of AD and enables investigation of the pathomechanisms of late-onset AD. Amyloid-β (Aβ) production, TAU phosphorylation, and GSK3B activation were investigated in all lines. Moreover, cellular responses to extracellular hydrogen peroxide and Aβ_1–42_ oligomers were analyzed in control and AD-derived neural cells. We demonstrate that both fAD and sAD pathology can be recapitulated and that TAU pathology can be investigated in an iPSC-based in vitro human cellular model.

## Methods

The chemicals used were purchased from Sigma-Aldrich (St. Louis, MO, USA), and the cell culture reagents and culture plates were purchased from Thermo Fisher Scientific (Waltham, MA, USA), unless specified otherwise.

### iPSC lines

iPSC lines derived from patients with AD used in this study were characterized and published earlier [41–46], as detailed in Table 1. The patients were clinically diagnosed and characterized by the Institute of Genomic Medicine and Rare Disorders, Semmelweis University, Budapest, Hungary, or at the Danish Dementia Research Centre, Rigshospitalet, University of Copenhagen, as described previously. Volunteers without dementia (assessed by clinical evaluation) were used as control subjects, from whom iPSC lines were established, characterized, and maintained under identical conditions as the AD iPSC lines. The hiPSC lines were maintained on Matrigel (BD Matrigel; STEMCELL Technologies, Vancouver, BC, Canada) in mTESR1 (STEMCELL Technologies) culture media. The media were changed daily, and the cells were passaged every 5–7 days using Gentle Cell Dissociation Reagent (STEMCELL Technologies) according to the manufacturer’s instructions.

**Table 1:** Cell lines used in this study

iPSC line name	Clone number	Identifier in the study	Disease	Mutation	Sex	Reference
BIOT-7183-PSEN1	S1	fAD-1	Early-onset fAD	*PSEN1* c.265G>C, p.V89L	F	[41]
	S2	fAD-2				
H234	C5	fAD-3	Early-onset fAD	*PSEN1* c.449T>C, p.L150P	M	[42]
H235	C6	fAD-4	Early-onset fAD	*PSEN1* c.449T>C, p.L150P	M	–
BIOT-0904-LOAD	S2	sAD-1	Late-onset sAD	Unknown	M	[43]
	S3	sAD-2				
BIOT-0630-LOAD	S4	sAD-3	Late-onset sAD	Unknown	F	[44]
BIOT-4828-LOAD	S1	sAD-4	Late-onset sAD	Unknown	F	[45]
	S6	sAD-5				
BIOT-0726-LOAD	S3	sAD-6	Late-onset sAD	Unknown	F	[46]
CTRL1	S9	Ctrl-1	Healthy	–	F	–
	S11	Ctrl-2				
H250	C16	Ctrl-3	Healthy	–	F	–
H256	C6	Ctrl-4	Healthy	–	M	–

*Abbreviations: iPSC* Induced pluripotent stem cell, *LOAD* Late-onset Alzheimer’s disease

### Neural induction of iPSCs

Neural progenitor cells (NPCs) were generated from each of the human iPSCs by dual inhibition of SMAD signaling pathway using LDN193189 and SB431542 [47]. Neural induction was initiated upon reaching a desired confluence of iPSCs on Matrigel-coated dishes by addition of neural induction medium (NIM) (1:1 vol/vol mixture of DMEM/F12 and neurobasal medium, 1× N-2 supplement, 1× B-27 supplement, 1× nonessential amino acids [NEAA], 2 mM l-glutamine, 50 U/ml penicillin/streptomycin, 100 μM β-mercaptoethanol, 5 μg/ml insulin), which was supplemented with 5 ng/ml basic fibroblast growth factor (bFGF), 0.2 μM LDN193189 (Selleckchem, Houston, TX, USA), and 10 μM SB431542. The NIM was changed every day. At day 10, neural rosettes were picked manually, replated on poly-l-ornithine/laminin (POL/L) (Sigma-Aldrich)-coated dishes, and expanded in neural maintenance medium (NMM) (1:1 vol/vol mixture of DMEM/F12 and neurobasal medium, 1× N-2 supplement, 1× B-27 supplement, 1× NEAA, 2 mM l-glutamine, 50 U/ml penicillin/streptomycin), and supplemented with 10 ng/ml epidermal growth factor and 10 ng/ml bFGF.

### Neural differentiation of NPCs

To generate human neurons, NPCs were plated on the POL/L-coated dishes and cultured in neural differentiation medium (1:1 vol/vol mixture of DMEM/F12 and neurobasal-A medium, 1× N-2 supplement, 1× B-27 supplement, 1× NEAA, 2 mM l-glutamine, 50 U/ml penicillin/streptomycin) supplemented with 0.2 mM ascorbic acid and 25 μM β-mercaptoethanol. For terminal differentiation (TD) into cortical neurons, the cells were plated on POL/L (0.002%/2 μg/cm^2^) at a seeding density of 40,000 cells/cm^2^ for immunocytochemistry (ICC) and 100,000 cells/cm^2^ for enzyme-linked immunosorbent assay (ELISA) and Western blotting experiments with NMM. The medium was changed every 3–4 days during the course of TD. The efficiency of TD was monitored by ICC staining and RT-qPCR for tubulin-β 3 class III (TUBB3) and microtubule-associated protein 2 (MAP2) expression at week 10. In the present study, NPCs from passage 9 to passage 10 were differentiated up to 10 weeks for ELISA and Western blotting experiments, whereas samples were collected at weekly intervals.

### Immunocytochemistry

Cells were fixed in 4% paraformaldehyde (PFA) for 20 minutes at room temperature (RT), washed twice with PBS, and permeabilized with 0.2% Triton X-100 in PBS for 20 minutes. Then, cells were blocked with 3% bovine serum albumin (BSA) in the presence of 0.2% Triton X-100 in PBS for 60 minutes at RT. The respective primary antibodies were applied overnight at 4 °C (Additional file 1: Table S1). To detect the signal, cells were incubated for 60 minutes at RT with the appropriate secondary antibodies (Alexa Fluor 488 donkey anti-rabbit immunoglobulin G [IgG] [H + L], Alexa Fluor 594 donkey anti-mouse IgG [H + L], Alexa Fluor 488 donkey anti-mouse IgG [H + L], or Alexa Fluor 594 donkey anti-rabbit IgG [H + L]; Life Technologies, Carlsbad, CA, USA). Cell nuclei were visualized using VECTASHIELD Mounting Medium with 4′,6-diamidino-2-phenylindole (1.5 μg/ml; Vector Laboratories, Burlingame, CA, USA). Cells were analyzed under a fluorescence microscope equipped with a 3D imaging module (Axio Imager system with ApoTome; Carl Zeiss MicroImaging GmbH, Göttingen, Germany) controlled using AxioVision 4.8.1 software (Carl Zeiss MicroImaging GmbH).

### Electron microscopy

To evaluate the neuronal cultures, a monolayer of 5-week-old neurons grown on POL/L-treated glass coverslips was fixed with a fixative solution containing 3.2% PFA, 0.2% glutaraldehyde, 1% sucrose, and 40 mM CaCl_2_ in 0.1 M cacodylate buffer for 24 h at 4 °C. Samples were rinsed for 2 days in cacodylate buffer, then postfixed in 1% ferrocyanide-reduced osmium tetroxide [48] for 1 h at RT. The samples were then treated with aqueous 1% uranyl-acetate for 30 minutes and embedded in Spurr low-viscosity epoxy resin medium (Sigma-Aldrich) according to the manufacturer’s instructions and cured for 24 h at 80 °C. Ultrathin sections were stained with Reynolds lead citrate for 2 minutes and examined under a JEOL JEM 1011 transmission electron microscope (JEOL, Peabody, MA, USA) operating at 60 kV. Photographs were taken using an Olympus Morada 11-megapixel camera and iTEM software (Olympus, Center Valley, PA, USA).

### Flow cytometry

NPCs growing on POL/L-coated dishes were dissociated into single cells with Accutase (Sigma-Aldrich) and fixed with 4% PFA for 20 minutes at RT. Cells were permeabilized with 0.2% Triton X-100 in PBS for 5 minutes at RT and blocked with 10% FBS in PBS for 20 minutes at RT. Cells were stained for 1 h at RT with Alexa Fluor 647 mouse anti-Nestin and phycoerythrin mouse anti-human paired box 6 (PAX6) antibodies (BD Pharmingen, San Jose, CA, USA). Flow cytometric analysis was performed using a Cytomics FC 500 flow cytometer (Beckman Coulter, Brea, CA, USA). To detect Nestin (NES) and PAX6 expression in NPCs, an argon laser (488 nm) and a red solid laser (635 nm), respectively, were used. Flow cytometric data analysis was performed using FlowJo software (version 7.6.5; FlowJo, LLC, Ashland, OR, USA).

### Immunoblotting

The cell cultures were lysed with RIPA Lysis and Extraction Buffer supplemented with Halt™ Protease and Phosphatase Inhibitor Cocktail and Pierce™ Universal Nuclease for Cell Lysis (all from Thermo Fisher Scientific). The protein extracts were derived from one well of a six-well plate of a single neuronal differentiation. Lysed samples were sonicated and incubated for 60 minutes on ice. Total protein concentration was determined using a Pierce BCA Protein Assay Kit. Cell lysates (2 μg) were separated on 10% sodium dodecyl sulfate-polyacrylamide gel and transferred to Immun-Blot® PVDF Membrane (Bio-Rad Laboratories, Hercules, CA, USA). The membranes were blocked with Tris-buffered saline with Tween 20 (TBST; 20 mM Tris-HCl, pH 7.4, 150 mM NaCl, 0.1% Tween 20) containing 5% BSA and then incubated with the respective primary antibody solution overnight at 4 °C (Additional file 1: Table S1). After being washed with TBST, the membranes were incubated with horseradish peroxidase-conjugated secondary antibodies for 1 h at RT. All the secondary antibodies were either goat anti-mouse or goat anti-rabbit (Sigma-Aldrich). All samples were analyzed at days 14, 28, 42, 56, and 70 of TD, and the amount of pTAU and pSer9GSK3B relative to total TAU and GSK3B in lysates was measured. Signals were detected with SuperSignal™ West Dura Extended Duration Substrate by KODAK Gel Logic 1500 Imaging System (Bruker, Billerica, MA, USA) and Kodak MI SE imaging software (Carestream, Atlanta, GA, USA). Densitometric measurement of protein band intensity was carried out using Image Studio™ Lite software (LI-COR Biosciences, Lincoln, NE, USA). Phosphorylation of TAU and GSK3B protein was calculated from densitometric data of phosphoproteins divided by densitometric data of nonphosphorylated proteins.

### Measurement of Aβ_1–40_ and Aβ_1–42_ by ELISA

Conditioned medium was collected after 4 days of culture (without media change) at every week from one well of a six-well plate. To prevent protein degradation, 4-(2-aminomethyl)benzenesulfonyl fluoride hydrochloride was added to the medium. Extracellular Aβ_1–40_ and Aβ_1–42_ levels were measured using the Human β-Amyloid (1-40) ELISA Kit and Human β-Amyloid (1-42) ELISA Kit (Wako Chemicals, Neuss, Germany) according to the manufacturer’s instructions. The secreted Aβ levels determined (in picomolar concentrations) were normalized to total protein content of cell lysate. The signal was detected using a Varioskan Flash multimode reader (Thermo Fisher Scientific).

As a control value we used the average value (±SEM) of the four clones derived from healthy individuals (ctrl-1, ctrl-2, ctrl-3, ctrl-4) in all experiments. The individual expression levels are presented in Additional file 2: Figure S1.

### RT-qPCR

Total RNA was isolated from differentiated neurons at different time points using the RNeasy Plus Mini Kit (QIAGEN, Valencia, CA, USA) according to the manufacturer’s protocol. One microgram of RNA was transcribed using the SuperScript™ III VILO™ cDNA Synthesis Kit (Thermo Fisher Scientific). The PCR conditions were subjected to 94 °C for 3 minutes as an initial denaturation step, followed by 40 cycles of 95 °C for 5 seconds for denaturation, 60 °C for 15 seconds for annealing, and 72 °C for 30 seconds for elongation. The amplification reactions were carried out in a total volume of 15 μl using SYBR Green JumpStart Taq ReadyMix (Sigma-Aldrich). RT-qPCR was run on the Rotor-Gene Q 5plex Platform (QIAGEN) using oligonucleotide primers detailed in Additional file 3: Table S2. Human glyceraldehyde 3-phosphate dehydrogenase *GAPDH*) was used as a reference gene. The data were analyzed using REST software (2009; version 2.0.13).

### Library preparation and next-generation sequencing

Genomic DNA (gDNA) was isolated from peripheral blood mononuclear cells using the GenElute Mammalian Genomic DNA Miniprep Kit (Sigma-Aldrich). An ND-1000 spectrophotometer (NanoDrop Technologies, Wilmington, DE, USA) was used to determine the concentration and purity of the isolated gDNA. The gDNA integrity was evaluated by 0.8% agarose gel electrophoresis.

The multiplexed library preparation was performed by using the SureSelect^QXT^ and SureSelect^XT^ Human All Exon V6 Target Enrichment systems according to the manufacturer’s instructions (Agilent Technologies, Santa Clara, CA, USA). The probe set targeted > 99% of RefSeq (~20,000) genes with a total target size of 60 Mb. Pooled libraries were sequenced on the NextSeq 500 platform (Illumina, San Diego, CA, USA) in a 2 × 80-bp paired-end run. The average throughput was > 80 million reads (4 Gbp per sample). Primary data analysis, filtering, and adapter trimming were performed using NextSeq Control Software (version 1.4.1; Illumina). Alignment was done using the Burrows-Wheeler Aligner (Illumina) [49] to the GRCh37/hg19 reference genome, and variant calling was performed with the Genome Analysis Toolkit (GATK) [50]. Variants were annotated using the wANNOVAR web server [51, 52], and their phenotypic effects were evaluated using the American College of Medical Genetics and Genomics standards and guidelines [53].

### Cell viability assay after H_2_O_2_ and Aβ treatment

Neurons growing on a 96-well plate at days 28 and 56 of TD were treated with 30 μM and 60 μM of hydrogen peroxide (H_2_O_2_) (Sigma-Aldrich) or 5 μM Aβ_1–42_ oligomer solution. For preparation of oligomer solution, iso-Aβ_1–42_ peptides were used as detailed elsewhere [54] (provided by University of Szeged, Hungary). Viability of the cultures following H_2_O_2_ and Aβ treatment was assessed at 24 h of stimulation using a CellTiter-Glo® Luminescent Cell Viability Assay (Promega, Madison, WI, USA). The luminescent signal was detected using the Varioskan Flash Multimode Reader. Neuronal survival was represented as a percentage of control.

### Statistical analysis

All results were analyzed using Prism 5 (GraphPad Software, La Jolla, CA, USA) and Microsoft Office 2010 (Microsoft, Redmond, WA, USA) software. All data on the graphs represent the average of the triplicate measurements (*n* = 3). The “*n*” value corresponds to the number of replicates for each cell line. Analysis of data is presented as the mean ± SEM. Dunnett’s method was used to compare the individual groups with control subjects. In all cases, significance was noted at *p* < 0.05.

## Results

### Neuronal differentiation capacity of iPSCs derived from patients with fAD and patients with sAD is similar

Samples from patients with fAD and patients with sAD were reprogrammed, and iPSC lines were generated and characterized by our teams as previously reported [41–46]. In the present study, we studied iPSC clones derived from three patients with fAD with mutation in *PSEN1* (contributing with in total four lines: fAD-1, fAD-2, fAD-3, fAD-4), four patients with late-onset sAD (contributing with in total six lines: sAD-1, sAD-2, sAD-3, sAD-4, sAD-5, sAD-6), and three individuals without dementia as control subjects (contributing with in total four lines: ctrl-1, ctrl-2, ctrl-3, ctrl-4) (*see also* Table 1). In all sAD samples, next generation sequencing analysis revealed a lack of pathogenic mutations in *APP*, *PSEN1*, and *PSEN2*, indicating late-onset AD. Moreover, sequencing of *MAPT* and *GRN* excluded FTD as well. The APOE status of the cell lines included in the study is shown in Additional file 4: Table S3.

**Fig. 1:**
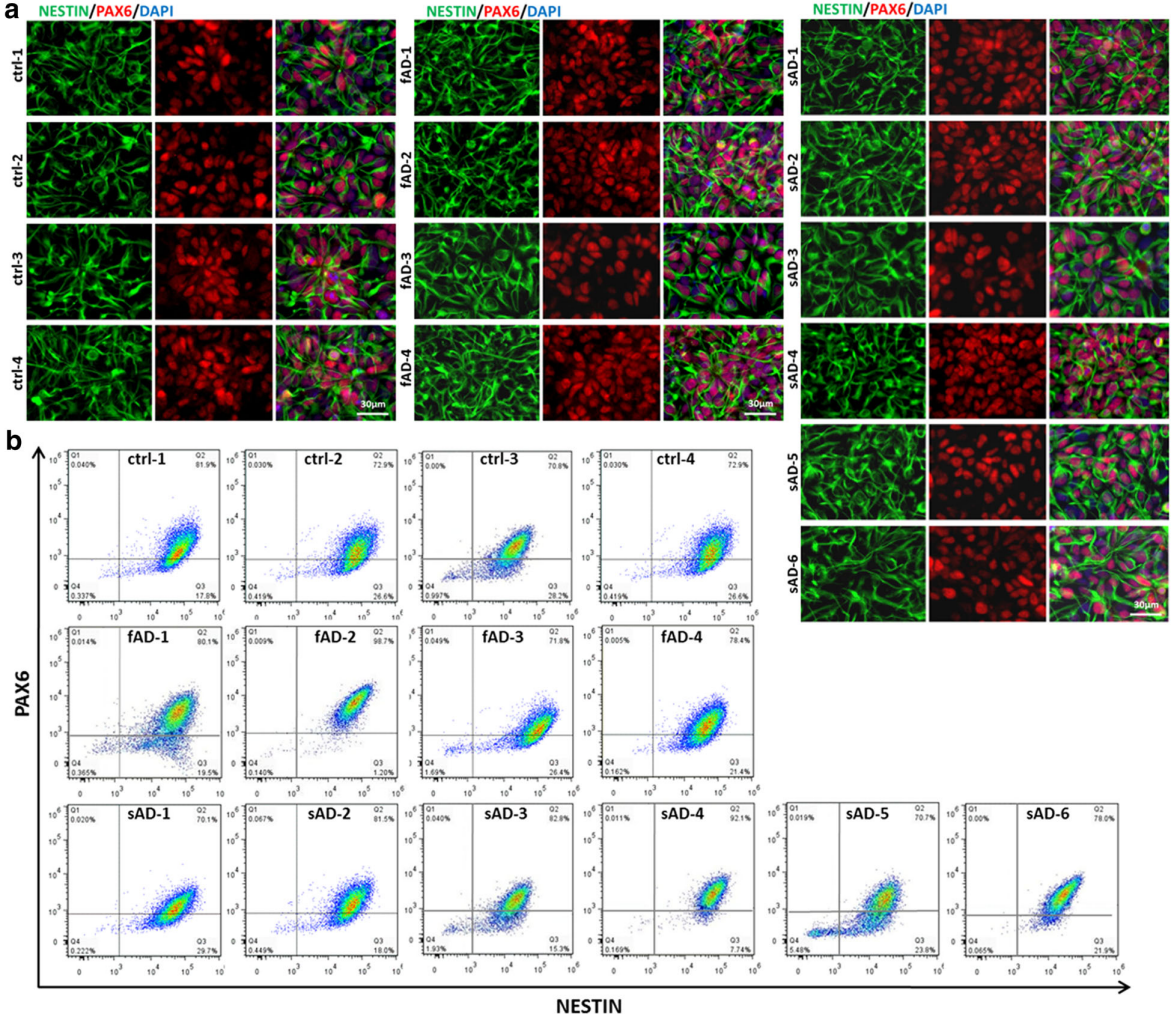
Immunofluorescence characterization of the induced pluripotent stem cells (iPSCs) derived neural progenitor cells. **a** Representative images of neural progenitor cells (NPCs) from healthy control individuals (ctrl-1–ctrl-4), patients with early-onset familial Alzheimer’s disease (fAD-1–fAD-4), and patients with late-onset sporadic Alzheimer’s disease (sAD-1–sAD-6) stained with Nestin (*green*), paired box 6 (PAX6; *red*), and 4′,6-diamidino-2-phenylindole (DAPI; *blue*). All three groups exhibited similar progenitor marker expression. scale bar = 30 μm. **b** Flow cytometric analysis of cultured NPCs. Dot plots demonstrate similar expression pattern of PAX6 and Nestin in control, fAD, and sAD iPSC line-derived NPC cultures with similar passage numbers (p6)

All AD iPSCs and control iPSCs were successfully converted into neuronal progenitor cells (NPCs) and expressed neuroepithelial markers PAX6 and NES (Fig. 1a). To quantify the efficiency of NPC formation, flow cytometry was applied, which showed that there were no significant differences between any of the cell lines with respect to generating NPCs including > 70% copositivity for PAX6 and NES (Fig. 1b).

**Fig. 2:**
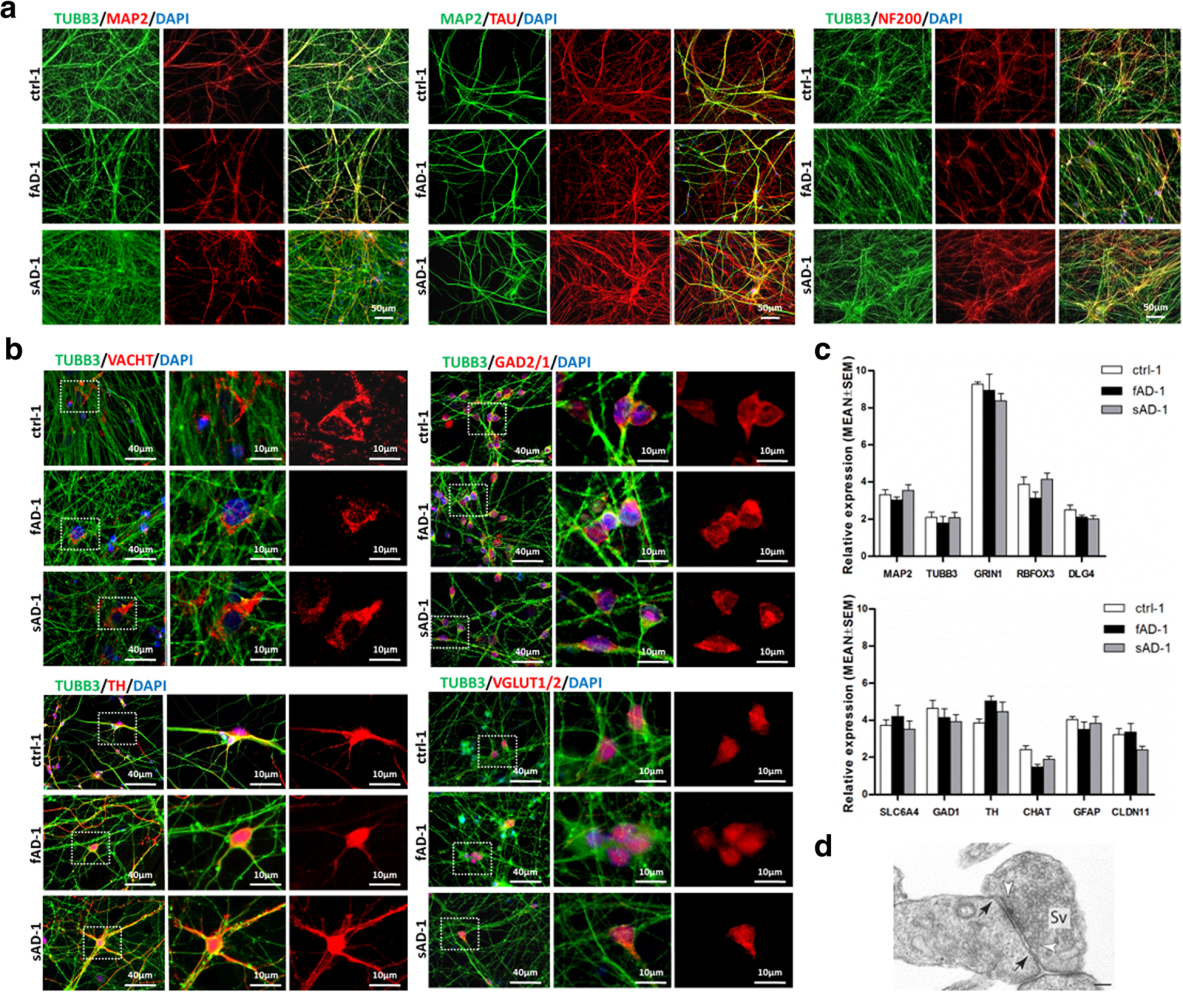
Neuronal differentiation from control and Alzheimer’s disease induced pluripotent stem cells (AD-iPSCs). **a** Representative immunofluorescence images show expression of neuronal markers at day 70 of terminal differentiation (TD70): tubulin β 3 class III (TUBB3; *green*, *left panel*), microtubule-associated protein 2 (MAP2; *red*, *left panel*), TAU (*red*, *middle panel*), and neurofilament, heavy polypeptide 200 kDa (NF200; *red*, *right panel*). Scale bar = 50 μm. **b** Differentiation of iPSCs into various neuronal subtypes at TD70 was confirmed by the presence of specific markers: vesicular acetylcholine transporter (VACHT) (cholinergic neurons), glutamic acid decarboxylases 2 and 1 (GAD2/1) (GABAergic neurons), TH (dopaminergic neurons), and vesicular glutamate transporter 1/2 (VGLUT1/2) (glutamatergic neurons). Scale bars = 40 μm and 10 μm as indicated. **c** Gene expression of neuronal markers from ctr-1, early-onset familial Alzheimer’s disease (fAD-1), and sporadic Alzheimer’s disease (sAD-1) lines at TD70 obtained by qPCR. The expression values were normalized to *GAPDH* and calculated as a relative amount of messenger RNA versus expression value of neural progenitor cells, which was set to 1. Data are reported as mean ± SEM of three independent measurements. AD-iPSC neurons demonstrate expression pattern similar to that of neuronal cells derived from control iPSCs. **d** Ultrastructure of a synapse at TD35 with synaptic vesicles (Sv) and synaptic cleft with synaptic junctions (tight junctions, *black arrowheads*). Docking synaptic vesicles are also observable (*white arrowheads*). Scale bar = 100 nm. *DAPI* 4′,6-Diamidino-2-phenylindole

Next, the neuronal differentiation of fAD and sAD NPCs was investigated and compared with that of control lines at different time points (TD14, TD28, TD42, TD56, TD70). ICC labeling showed that the increasing expression of neuronal markers TUBB3 and MAP2 during neuronal differentiation was comparable among AD and control cell lines (Fig. 2a). More mature markers—TAU and neurofilament, heavy polypeptide 200 kDa (NF200)—appeared later during differentiation than MAP2 and TUBB3 (Fig. 2a). ICC analysis at day 70 of AD and control neural cultures revealed the presence of proteins related to distinct subtypes of mature neurons: markers of cholinergic neurons (vesicular acetylcholine transporter [VACHT]), dopaminergic neurons (tyrosine hydroxylase [TH]), GABAergic (glutamic acid decarboxylases 2 and 1 [GAD2/GAD1]), and glutamatergic neurons (vesicular glutamate transporter 1/2 [VGLUT1/2]) (Fig. 2b). In order to compare gene expression levels, RT-qPCR was performed on day 70 neurons. The analysis revealed that the AD and control lines expressed comparable levels of *MAP2*, sodium-dependent serotonin transporter (*SLC6A4*), NMDA receptor subunit NR1 (glutamate ionotropic receptor *N*-methyl-d-aspartate type subunit 1 [*GRIN1*]), marker of maturing neurons – RNA binding protein, fox-1 homolog 3 (*RBFOX3*), and *GAD1* (Fig. 2c). Moreover, they also showed high expression levels of dopaminergic neuron marker (*TH*), astrocyte marker (glial fibrillary acidic protein [*GFAP*]), and an early oligodendrocyte marker (claudin 11 [*CLDN11*]) (Fig. 2c). Furthermore, the cells expressed *TUBB3*, choline *O*-acetyltransferase (*CHAT*), and postsynaptic density protein 95 (discs large MAGUK scaffold protein 4 [*DLG4*]). Importantly, no evident differences in gene expression levels were observed between control, fAD, and sAD lines. Additionally, electron microscopy of neural cell cultures at day 35 revealed the presence of synapses with docking of synaptic vesicles and synaptic clefts (Fig. 2d). These findings indicate that AD has no significant effect on the overall neural differentiation of iPSCs in the used system, as well as that NPCs can be differentiated into various neuronal and glial cell types with similar efficiency independent of their origin from patients with AD or healthy individuals.

### Elevated level of Aβ was secreted by fAD and sAD neurons

**Fig. 3:**
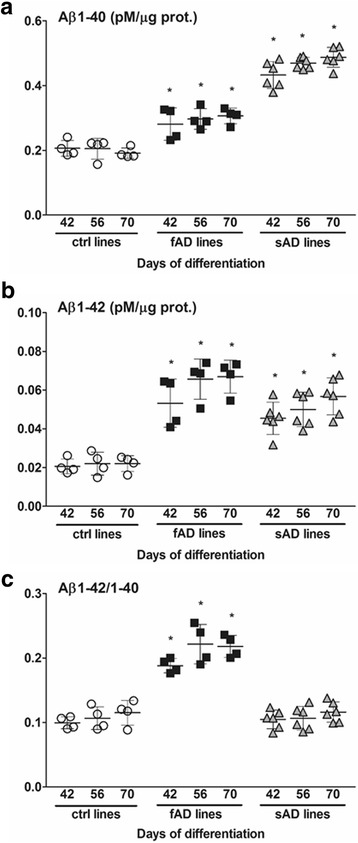
Characterization of amyloid-β (Aβ) secretion in control and Alzheimer’s disease induced pluripotent stem cell (AD iPSC)-derived neurons at terminal differentiation day 42 (TD42), TD56, and TD70. **a** The amount of secreted Aβ_1–40_ from control-, early-onset familial Alzheimer’s disease (fAD)- (fAD-1–fAD-4), and sporadic Alzheimer’s disease (sAD)- (sAD-1–sAD-6)-iPSC-derived neurons. **b** The amount of Aβ_1–42_ released from control- and AD-iPSC-derived neurons. **c** The ratio of Aβ_1–42_/Aβ_1–40_ from neurons derived from control and AD lines. Aβ_1–40_ and Aβ_1–42_ secreted from neural cells into the medium were measured at day 4 after the last medium change. The extracellular Aβ levels determined (in picomolar concentrations) were normalized to total protein content of cell lysates. Data represent mean ± SEM (*n* = 3). Dunnett’s test was performed to evaluate the significance of groups compared with control at the same time point of differentiation (*p < 0.05)

To determine Aβ production from iPSC lines of patients with fAD and patients with sAD, secreted Aβ_1–40_ and Aβ_1–42_ levels were measured weekly by ELISA in the conditioned media of differentiated neural cell lines from the second to tenth weeks of differentiation. Elevated secreted Aβ_1–40_ and highly increased Aβ_1–42_ levels were observed in all fAD neural cultures compared with the mean level in the control lines at almost all time points (Fig. 3). The Aβ_1–42_/Aβ_1–40_ ratio was also increased up to twofold for the fAD-iPSC-derived neurons. The extracellular level of Aβ_1–40_ in all sAD neurons was significantly higher than in control and fAD neurons. The Aβ_1–42_ secretion in sAD neurons was also elevated compared with the average values of the control neurons; however, the levels did not reach the same levels as in fAD neurons. Interestingly, the measured Aβ_1–42_/Aβ_1–40_ ratio in all sAD-iPSC-derived neurons was similar to that in control neurons (Fig. 3c). In nonmature neurons up to day 14 (TD14), Aβ production was comparable among AD and control lines (Additional file 2: Figure S1). During neural differentiation, Aβ_1–40_ and Aβ_1–42_ levels gradually increased to reach the investigated maximum at day 70 of TD (endpoint of experiment; TD70), suggesting a maturation-dependent secretion of Aβ (Fig. 3, Additional file 2: Figure S1). The results at TD14 and TD28 indicated a transition period in which not all AD samples showed significant differences from the control samples, but from TD42, all AD samples displayed elevated amounts of Aβ. Therefore, we concentrated our further experimental analyses on the samples matured for a minimum of 6 weeks.

**Fig. 4:**
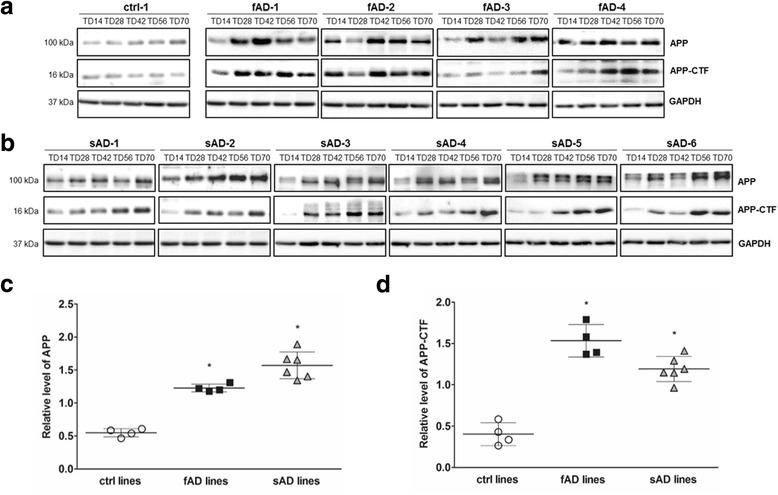
Characterization of amyloid precursor protein (APP) and amyloid precursor protein carboxy-terminal fragment (APP-CTF) expression during neuronal differentiation. Representative immunoblots of (**a**) APP and APP-CTF in control neurons (ctrl-1), early-onset familial Alzheimer’s disease (fAD)-derived neurons (fAD-1–fAD-4), and (**b**) sporadic Alzheimer’s disease (sAD)-derived neurons (sAD-1–sAD-6) at terminal differentiation day 14 (TD14), TD28, TD42, TD56, and TD70. Quantification of (**c**) APP and (**d**) APP-CTF signals at TD70 was normalized to *GAPDH*. Data are presented as mean ± SEM (*n* = 3). Dunnett’s test was performed to evaluate the significance of groups compared with control (*p < 0.05)

To evaluate the level of APP, the direct precursor of Aβ in neurons, we performed Western blot analysis. The results revealed that APP expression increased during neuronal differentiation and reached a maximum at day 70 (TD70) in all fAD and sAD neural cultures (Fig. 4, Additional file 5: Figure S2a). Expression of this protein was significantly increased in all AD cell lines from TD42. Subsequently, we quantified the amount of amyloid precursor protein carboxy-terminal fragment (APP-CTF) generated through APP cleavage by β-secretase. As shown in Fig. 4 and Additional file 5: Figure S2b, remarkably higher APP-CTF protein levels were detected in fAD and sAD neurons. No significant differences in the expression of APP and APP-CTF were observed between fAD and sAD neurons. In summary, both Aβ and APP levels were significantly elevated in fAD and sAD neural cultures compared with control cultures. These differences were detectable after 6 weeks (TD42) but were prominent with all sAD lines at week 10 (TD70).

### Epitope-specific TAU hyperphosphorylation was detected in fAD and sAD lines

**Fig. 5:**
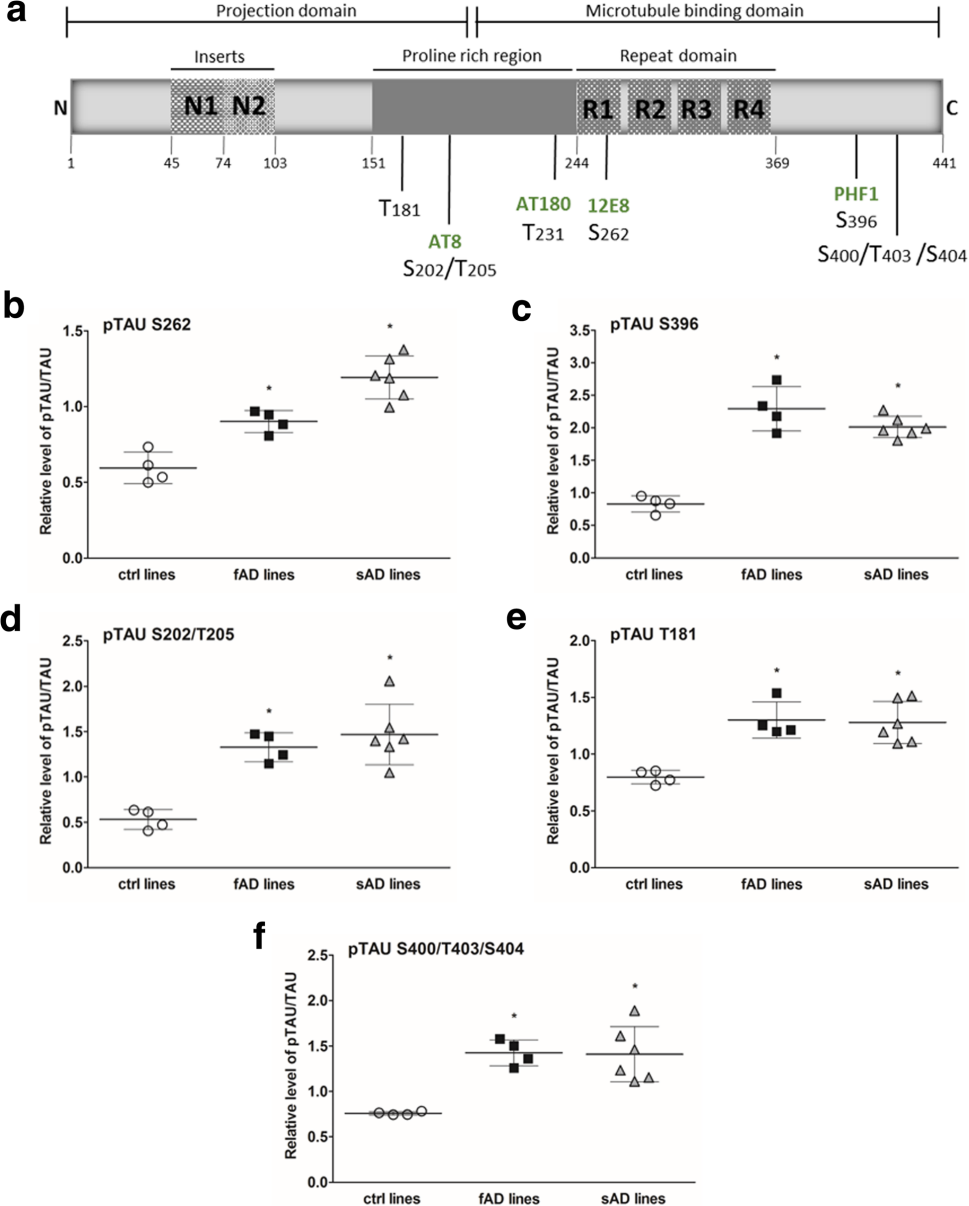
Western blot analysis of total TAU and phosphorylated TAU (pTAU) protein. **a** Schematic representation of human TAU isoform (441 amino acids) with the functional projection and microtubule-binding domains. Projection domains including a proline-rich region and N-terminal part interact with cytoskeletal elements and are involved in signal transduction. Microtubule-binding domains with a C-terminal part regulate the microtubule polymerization and bind to proteins such as presenilin 1 (PSEN1). Epitopes of pTAU antibodies analyzed in this study are indicated on the scheme. **b**–**f** Densitometric analysis of TAU phosphorylated at different epitopes: S262, S396, S202/T205, T181, and S400/T403/S404. All samples were analyzed at day 70 of terminal differentiation. The amount of pTAU relative to total TAU levels in the lysates was measured. *GAPDH* as the loading control was used to normalize the data. All values are the mean ± SEM (*n* = 3). Dunnett’s test was performed to evaluate the significance of groups compared with control (*p < 0.05)

One of the proposed mechanisms for the TAU protein pathomechanism in AD are posttranslational modifications through abnormal phosphorylation (hyperphosphorylation) (reviewed in [55]). The appearance and accumulation of abnormally phosphorylated TAU leads to mislocalization and aggregation of TAU as well as formation of NFTs [56]. Therefore, we focused on the alterations of TAU phosphorylation in our fAD and sAD neural cultures. TAU protein contains several phosphorylation sites (reviewed in [57]). We examined six different phosphorylation sites (Fig. 5a) in fAD, sAD, and control neural cultures at different time points of TD. We observed significantly higher phosphorylation of TAU at Ser262 (12E8 epitope) in all our AD neurons than in control neurons (Fig. 5b, Additional file 6: Figure S3a). Elevated phosphorylation was detected already at TD42 and reached the highest level between weeks 8 and 10 (TD56 and TD70) of TD. Further analysis revealed upregulated TAU phosphorylation at the Ser396 epitope in all fAD and sAD neural cultures (Fig. 5c, Additional file 6: Figure S3b). Western blot quantification showed an increase in TAU phosphorylation at Ser202/Thr205 (AT8 epitope) in the AD group (Fig. 5d, Additional file 6: Figure S3c). Furthermore, we observed higher phosphorylation of TAU at Thr181 and Ser400/Thr403/Ser404 in both fAD and sAD neurons (Fig. 5e and f, Additional file 6: Figure S3d and e). On the basis of these results, we can conclude that, although the phosphorylation level showed some clonal variations, the ratio of pTAU/TAU was significantly elevated in all AD neurons and that no major differences were observed between fAD- and sAD-derived neurons.

### GSK3B activation in AD-derived neurons

**Fig. 6:**
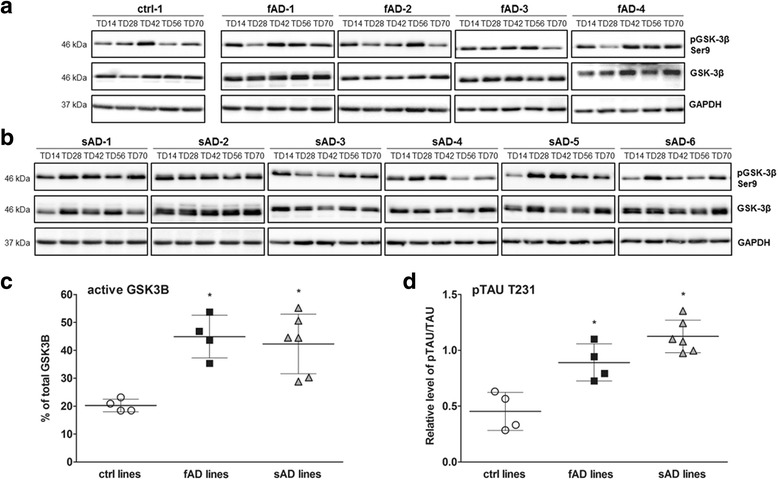
Analysis of glycogen synthase kinase 3β (GSK3B) and the active form of GSK3B in neuronal culture. **a** Representative immunoblotting shows the phosphorylation of GSK3B at Ser9 (inactive form of the kinase [102]) and GSK3B in control neurons (ctrl-1), early-onset familial Alzheimer’s disease (fAD) neurons (fAD-1–fAD-4), and (**b**) sporadic Alzheimer’s disease (sAD) neurons (sAD-1–sAD-6) at all time points of neuronal terminal differentiation (TD14–TD70). **c** Quantification of the active GSK3B form at TD70 was presented as a percentage of nonphosphorylated GSK3B at Ser9. **d** Densitometric analysis of TAU phosphorylated at the T231 epitope at TD70. All values were normalized to *GAPDH* and are presented as mean ± SEM (*n* = 3). Dunnett’s test was performed to evaluate the significance of groups compared with control (*p < 0.05)

Scientific evidence suggests that GSK3B is involved in many pathological hallmarks of AD, including hyperphosphorylation of TAU [58], increased Aβ production [59], memory impairment, and neuronal loss [60]. To verify if AD neurons with elevated TAU phosphorylation have increased GSK3B activity, the percentage of the active form of GSK3B was calculated by assessing the ratio of inhibitory phosphorylation on Ser9 by immunoblotting. Our results demonstrate that fAD and sAD neurons in most cases exhibited significantly higher levels of active GSK3B than control neurons (Fig. 6a–c, Additional file 7: Figure S4a). During neuronal differentiation, the level of active GSK3B in control neurons remained constant, whereas in fAD and sAD neurons, the active GSK3B amount increased to reach its maximum at day 56 (sAD-2) or day 70 of TD for all remaining cell lines (Fig. 6c, Additional file 7: Figure S4a). Furthermore, higher phosphorylation of TAU at Thr231 (AT180 epitope), which can be catalyzed by GSK3B in vitro [61], was observed in all fAD and sAD neurons (Fig. 6d, Additional file 7: Figure S4b). These results indicate that activation of the GSK3B signaling in both fAD and sAD neurons contributes to the abnormal TAU phosphorylation pattern observed in these cell lines.

### Oxidative stress response was strongly affected in fAD and sAD neuronal cultures

**Fig. 7:**
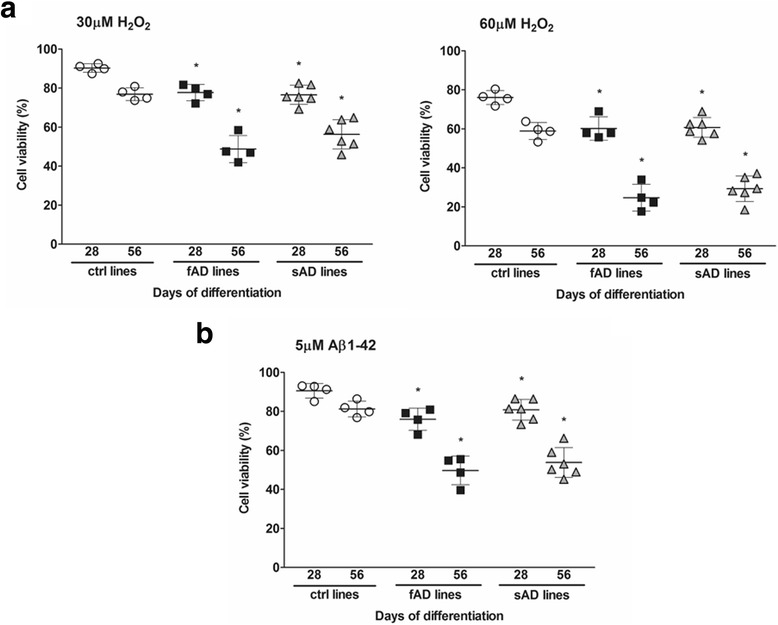
Effect of hydrogen peroxide (H_2_O_2_) and amyloid-β_1–42_ (Aβ_1–42_) oligomer treatment on neuronal viability. **a** Viability of induced pluripotent stem cell (iPSC)-derived neurons from control individuals (ctrl-1–ctrl-4), patients with early-onset familial Alzheimer’s disease (fAD) (fAD-1–fAD-4), and patients with sporadic Alzheimer’s disease (sAD) (sAD-1–sAD-6) at day 28 of terminal differentiation (TD28) and TD56 after 24 h of treatment with 30 μM H_2_O_2_ (*left panel*) and 60 μM H_2_O_2_ (*right panel*). **b** Neuronal survival of control and Alzheimer’s disease (AD) clones at TD28 and TD56 cultured 24 h in the presence of 5 μM Aβ_1–42_ oligomer solution. Neuronal survival was represented as a percentage of control. Viability of the cultures following H_2_O_2_ and Aβ treatment was assessed using the CellTiter-Glo® Luminescent Cell Viability Assay. Values are presented as the mean ± SEM (*n* = 3). Dunnett’s test was performed to evaluate the significance of groups compared with control at the same time point of differentiation (*p < 0.05)

Oxidative stress plays an important role in the pathogenesis of neurodegenerative disorders. The molecular mechanism of reactive oxygen species (ROS) action in the nervous system has been studied ([62, 63], reviewed in [64]). Despite this, the effect of oxidative stress on human iPSC-derived AD neurons is still not well studied. In our experiments, we used H_2_O_2_ in two different concentrations to examine neuronal response to a stressor. In AD and control neurons, treatment with increasing doses of H_2_O_2_ engendered a dose-dependent loss of cell viability; however, these two cell groups presented differential susceptibility to the stressor. The fAD and sAD neurons were more sensitive than control neurons to H_2_O_2_, and cell death caused by 30 μM and 60 μM H_2_O_2_ reached about 50% and 70%, respectively, in the AD cultures (Fig. 7a).

Evidence of the toxicity of Aβ to neurons has been demonstrated in rodent models and neuronal cell lines (reviewed in [65, 66]). Owing to the limited studies on neurons generated from iPSCs, we examined the effect of synthetic Aβ_1–42_ oligomer solution on iPSC-derived neurons. Cells treated at days 28 and 56 of neural differentiation for 24 h with 5 μM Aβ_1–42_ oligomers showed reduced neuronal survival, depending on the neuronal maturation state (Fig. 7b). Relative to control samples, AD cultures had enhanced sensitivity to cell toxicity induced by Aβ_1–42_. The 28-day-old AD neurons displayed about 15–20% cell death compared with 10% cell loss in control individuals. Mature neurons at TD56 were more susceptible to cell death; almost 40% of neurons died in AD cultures, whereas cell loss in control neurons increased slightly to 15% (Fig. 7b). On the basis of our results, we can conclude that fAD and sAD neural cultures are more susceptible to Aβ_1–42_ oligomer-induced cell death than the control cultures.

## Discussion

The present study shows that particular key disease phenotypes of the most common age-related neurological disorder, AD, can be modeled using patient-specific, iPSC-derived neural cells. Over the past few decades, primary neurons from animal models and immortalized neuronal cell lines have been used to study neurodegenerative diseases. Owing to many limitations, such as genetic and physiological differences between human and rodent brains, such studies of AD mechanisms have been controversial. Most animal models of AD are generated on the basis of modifications in three genes related to fAD (*APP*, *PSEN1*, and *PSEN2*), whereas modeling of sAD in rodents is currently not well examined. Therefore, there are huge expectations regarding iPSC technology, which allows for the generation of pluripotent cells from any individuals in the context of their own genetic identity, to provide new patient-specific in vitro disease models to study neurological disorders.

Modeling AD using iPSCs was initiated with fAD carrying mutations in *PSEN1*, *PSEN2*, and *APP*. Until now, only a few groups have reported generation of iPSC-derived neurons from patients with fAD, and sAD has been studied even less [36, 37, 40, 67]. In our present study, we analyzed samples and generated neurons from patients with fAD carrying pathogenic mutations in the *PSEN1* gene (V89L and L150P; these mutations were first identified by Queralt et al. [68] and Wallon et al. [69], respectively) and patients with sAD (Table 1). Our findings demonstrate that iPSCs derived from patients with fAD and patients with sAD can be successfully induced into NPCs with very uniform expression of NES and PAX6 and that they can be further differentiated into neurons and glial cells. Gene expression and ICC analysis revealed the presence of various neuronal subtypes, including GABAergic, glutamatergic, cholinergic, and dopaminergic neurons and progenitor cells of astrocytes and oligodendrocytes in control and AD cultures. We therefore conclude that there is no prominent difference in the differentiation and maturation propensity or in marker gene expression between control and AD neural cells, which is in accordance with a previous report [37].

Initially, our main question was to evaluate if sAD can be modeled through iPSC-derived neural cultures and represent a suitable model system for neuropathological investigations and drug development studies. Therefore, we first analyzed the relevant in vivo pathological hallmarks of the disease in our in vitro system. The accumulation of Aβ into extracellular aggregates is one of the pathological signs of AD in the human brain. Alterations in the level of Aβ peptides are often presented as the ratios between different isoforms. In *PSEN* mutant mouse models, elevated Aβ production can be detected [70–72]. In humans, elevated Aβ production was revealed in iPSC-derived neural lines from patients with fAD [36]. However, another study demonstrated that Aβ secretion in sAD-derived neurons is not consistently altered [37]. Our study demonstrated increased extracellular Aβ_1–40_ and Aβ_1–42_ levels in neurons derived from all fAD and sAD lines in a maturation-dependent manner. Interestingly, Aβ_1–40_ secretion was approximately twofold higher in sAD neurons than in fAD, whereas Aβ_1–42_ levels were similar. Moreover, we observed an elevated ratio of Aβ_1–42_ to Aβ_1–40_, one of the AD hallmarks, in fAD-derived neurons, whereas the Aβ_1–42_/Aβ_1–40_ ratio in sAD lines remained unchanged and comparable to that of non-AD control lines. Additionally, we observed upregulated expression of APP and APP-CTF in all AD-derived cell lines, which is in line with the increased Aβ levels we measured. Some groups also detected an increased level of Aβ_1–40_, Aβ_1–42_, and Aβ_1–42_/Aβ_1–40_ ratio in fAD cell lines with mutations in *PSEN1*, *PSEN2*, and *APP* [36, 37, 40]. On the basis of the above results, we can conclude that mutations in *PSEN1* may change the metabolism of Aβ peptides and drive amyloidosis in patients with fAD. Our findings also indicate the possible heterogeneity of fAD and sAD. AD-iPSC lines with *PSEN1* mutations and sAD do not always recapitulate the same phenotypes [37]. In fAD, genetic factors modify the clinical phenotype of the disease, whereas mechanisms underlying the pathogenesis of sAD are still not well understood and combine multiple genetic and environmental risk factors. It is possible that underlying mutations that have not yet been discovered may play an important role in sAD, reflecting the inherent variability of iPSCs. Thus, more cell lines have to be analyzed to reveal the broad heterogeneity of AD phenotypes.

We have demonstrated that another pathological hallmark characteristic of AD, TAU hyperphosphorylation, could be detected in AD neurons. Conformational changes and misfolded protein structure result in aberrant aggregation of TAU into neurofibrillary structures, NFTs [73]. TAU phosphorylation at various sites affects TAU activity, as well as its biological function and pathogenic role. Studies on the physiological properties of TAU have revealed that phosphorylation of Ser262 significantly diminishes the ability of TAU to bind microtubules [74]. Others have reported that phosphorylation of few KXGS motifs, including Ser262 and Ser356, reduces TAU binding capacity to microtubules and thus increases the dynamics of microtubules, which plays an important role in neurite growth and the development of neuronal polarity [75]. Phosphorylation at Thr231 leads to decreased ability of TAU to bind microtubules, reduces the level of acetylated tubulin, and consequently leads to microtubule destabilization [33]. Moreover, the increased TAU phosphorylation at Ser396 and Ser404 impairs microtubule assembly by detachment of TAU molecules from microtubules [76]. Elevated phosphorylation on Ser262, Thr231, and Ser396 residues can be detected early in the AD disease process [77, 78].

Quantitative in vitro data have revealed a negative impact of TAU phosphorylation at many epitopes on TAU activity and microtubule stability [31, 32, 79]. Previous studies on iPSC-derived, patient-specific fAD and sAD neurons have been limited to detecting mostly only one TAU phosphorylation site at Thr231 [40, 67]. We analyzed TAU phosphorylation in iPSC-derived neurons from patients with fAD and patients with sAD at six different phosphorylation sites. As a novel finding, we demonstrated increased TAU phosphorylation at all examined epitopes (Fig. 5a): Ser262, Ser202/Thr205, Ser396, Ser400/Thr403/Ser404, Thr181, and Thr231 in both fAD and sAD neurons. According to the literature, phosphorylation of TAU at Ser262 and Thr231 greatly diminishes its ability to bind microtubules, by 35% and 25%, respectively [74], whereas Ser396 and Ser404 phosphorylation generates more fibrillogenic TAU in vitro [78], which shows an increased propensity to aggregate [80]. Moreover, phosphorylation of the AT8 epitope results in a decrease in TAU microtubule nucleation activity, leading to microtubule depolymerization and destabilization [81]. TAU phosphorylation increased during neural differentiation, which is in agreement with in vivo studies on mice with *APP* mutations [82].

Furthermore, we have shown higher levels of active GSK3B in our AD cultures. This observation correlates with previously described findings for which researchers showed increased levels of active GSK3B measured in fAD and sAD neurons in vitro [40] and in transgenic animal models [83]. Pathological activation of GSK3B establishes a feedforward loop that contributes to abnormal APP processing [84], enhanced apoptosis in hippocampal neurons, TAU hyperphosphorylation, and synaptic failure in rodent models of AD [85, 86]. Our findings revealed that activation of GSK3B might contribute to TAU misregulation and abnormal phosphorylation. This is in accordance with previous reports confirming the important role of GSK3B in regulating TAU phosphorylation mostly on Thr231 and Ser199, Ser396, Ser400, Ser404, and Ser413 [87]. Consistent with this, restoring the normal level of GSK3B has been shown to reduce TAU hyperphosphorylation, decrease Aβ production and neuronal death in AD murine models [88], and decrease Aβ-induced neurotoxicity in cultured mouse primary neurons in vitro [89].

In our study, we evaluated cell viability after hydrogen peroxide and extracellular Aβ_1–42_ exposure in neurons at different maturation stages. Exposure to stress agents such as H_2_O_2_ induces ROS production and toxicity in many different cell types [90–92]. ROS show high reactivity with macromolecules and play an important role as signaling molecules (reviewed in [93]). Oxidative damage is linked with mitochondrial abnormalities and is catalyzed by the presence of Fe and Cu ions. In our results, we demonstrated a significant H_2_O_2_ dose-related decrease in the survival of fAD and sAD neurons. More mature neurons (TD56) showed a greater sensitivity to H_2_O_2_ than younger neuronal cultures (TD28). These observations indicate that H_2_O_2_ may provoke an antioxidant stress response resulting in increased level of ROS and may lead to subsequent cell death. Additionally, we observed that treatment with Aβ_1–42_ oligomers induced cell death in both fAD and sAD neurons. According to the literature, Aβ treatment may lead to activation of glutamate receptors and inhibition of glutamate transporters that leads to abnormal release of glutamate and disturbances in glutamatergic neurotransmission. Aβ treatment induces NMDA receptor-mediated cellular events in neurons and astrocytes, leading to synaptic damage and spine loss [94]. Chronic stimulation of NMDA receptor results in Ca^2+^ influx, which activates apoptotic pathways and increased glutamate excitotoxicity, leading to generation of ROS and to neuronal damage and cell death [95]. In cortical neurons, accumulation of glutamate and NMDA receptors promotes H_2_O_2_-mediated neurotoxicity and oxidative damage in DNA.

Furthermore, increased influx of Ca^2+^ mediated by Aβ treatment activates mitochondrial permeability transition pore, leading to deregulation of respiratory chain enzymes and ROS overproduction, and consequently to neurotoxicity [96]. On the basis of the above data, we can speculate that the neuronal death observed in our cultures upon synthetic Aβ treatment may be a consequence of mitochondrial stress and higher ROS production.

It has previously been reported that accumulated Aβ oligomers induce endoplasmic reticulum (ER) stress and ROS production [97], which lead to membrane lipid peroxidation and impairment of membrane protein function (reviewed in [98]). Furthermore, gene analysis of APP E693Δ neurons revealed upregulation of the oxidative stress-related markers [37]. Additionally, in vivo studies showed increased protein oxidation and lipid peroxidation in *PSEN1* mutant brains [99, 100], leading to destruction of spine morphology and impaired synaptic plasticity [101]. Thus, we speculate that our fAD and sAD neurons may also exhibit increased levels of stress-related gene expression, suggesting ER and Golgi abnormalities. It is worth considering that mutations in *PSEN1* and pathological changes in sAD, combined with neuronal aging, can upregulate ROS production, leading to mitochondrial damage that may contribute to neurodegenerative processes and AD progression.

Our results provide insight into the molecular basis of AD and provide patient cell models that are relevant for further AD investigations. In all fAD and sAD lines, we observed higher Aβ_1–40_ and Aβ_1–42_ secretion, increased activation of GSK3B, and hyperphosphorylation of TAU at six different epitopes. We showed that both fAD and sAD neurons revealed AD phenotypes, suggesting that sAD, with an unknown disease etiology, resembles the fAD phenotype.

The results of this study provide strong evidence that iPSC technology can be used in modeling the most common age-related neurodegenerative disorders, such as AD. This new strategy will make it possible to study pathological mechanisms on the molecular level and analyze patient-derived neurons in vitro from individuals who are still alive. Potentially, iPSCs from complex, unknown genetic background diseases, such as sAD, may be equally useful and able to provide suitable models such as the monogenic forms of AD carrying mutations in *PSEN1*, *PSEN2*, and *APP*.

Although iPSC-derived models afford the opportunity to identify factors associated with disease phenotypes, there are still many uncertainties that should be resolved in the near future. The most crucial limitations that must be addressed in future studies include development of reliable protocols for more rapid neuronal differentiation and optimization of culture conditions as well as current differentiation methods to eliminate clonal variability and heterogeneity of differentiated neuronal cells.

Several preclinical studies using fAD and sAD models have established new methods to gather insight into the molecular mechanisms, the role of different cell types, and patient-specific drug response. We believe that iPSCs have great potential for improving the model systems used in preclinical and clinical applications in neurodegenerative diseases.

## Conclusions

Our findings demonstrate that increased TAU phosphorylation occurs in fAD and sAD iPSC-derived mature neurons. To our knowledge, this is the first study involving comparison of TAU protein hyperphosphorylation in an iPSC-based fAD and sAD model. We also present evidence of elevated Aβ_1–40_ and Aβ_1–42_ levels in fAD and sAD samples; however, the Aβ_1–42_/Aβ_1–40_ ratios are significantly different from control cell lines in fAD cases, whereas the ratio of sAD cell lines not differ significantly. Moreover, we have shown the more sensitive oxidative stress response and higher susceptibility to exogenously added synthetic Aβ_1–42_ peptide solution of AD lines. Our findings demonstrate that the iPSC technology is suitable to model both fAD and sAD and may provide a platform for developing new treatment strategies for these conditions.

## Additional files

Additional file 1: Table S1. Antibodies used in this study. (TIF 141 kb)

Additional file 2: Figure S1. Characterization of Aβ secretion in control and AD iPSC-derived neurons at TD14 and TD28. **a** The amount of secreted Aβ_1–40_ and (**b**) Aβ_1–42_ from control-, fAD- (fAD-1–fAD-4), and sAD- (sAD-1–sAD-6)-iPSC-derived neurons. **c** The ratio of Aβ_1–42_/Aβ_1–40_ from neurons derived from control and AD lines. Aβ_1–40_ and Aβ_1–42_ secreted from neural cells into the medium were measured at day 4 after the last medium change. The extracellular Aβ levels determined (in picomolar concentrations) were normalized to total protein content. Data represent mean ± SEM (*n* = 3). Because the detected levels of Aβ_1–40_ and Aβ_1–42_ were not significantly different between the four healthy individual-derived clones (ctrl-1, ctrl-2, ctrl-3, ctrl-4), the average value (±SEM) as a control value was used in all graphs. Dunnett’s test was performed to evaluate the significance of groups compared with control (*p < 0.05). (TIF 396 kb)

Additional file 3: Table S2. PCR primers used in this study. (TIF 109 kb)

Additional file 4: Table S3. Polymorphism of AD- and FTD-related genes and APOE status of cell lines used in this study. Footnote: Cell lines not detailed in the table were not permitted to be involved in whole exome sequencing; therefore, only clinical mutation identification was available. (TIF 130 kb)

Additional file 5: Figure S2. Characterization of APP and APP-CTF expression during neuronal differentiation. Densitometric analysis of (**a**) APP and (**b**) APP-CTF expression in control neurons (ctrl-1), fAD-derived neurons (fAD-1–fAD-4), and sAD-derived neurons (sAD-1–sAD-6) at TD42, TD56, and TD70. Quantification of APP and APP-CTF signals was normalized to GAPDH. Data are presented as mean ± SEM (*n* = 3). Dunnett’s test was performed to evaluate the significance of groups compared with control (*p < 0.05). (TIF 1295 kb)

Additional file 6: Figure S3. Western blot analysis of total TAU and pTAU protein. **a–e** Densitometric analysis of TAU phosphorylated at different epitopes: S262, S396, S202/T205, T181, and S400/T403/S404. All samples were analyzed at days 42, 56, and 70 of terminal differentiation. The amount of pTAU relative to total TAU levels in the lysates was measured. GAPDH as the loading control was used to normalize the data. All values are the mean ± SEM (*n* = 3). Dunnett’s test was performed to evaluate the significance of groups compared with control (*p < 0.05). (TIF 804 kb)

Additional file 7: Figure S4. Analysis of GSK3B and active form of GSK3B in neuronal culture. **a** Quantification of active GSK3B form in control neurons (ctrl-1), fAD neurons (fAD-1–fAD-4), and sAD neurons (sAD-1–sAD-6) at days 42, 56, and 70 of terminal differentiation was presented as a percentage of nonphosphorylated GSK3B at Ser9 (inactive form of the kinase [102]). **b** Densitometric analysis of TAU phosphorylated at T231 epitope at days 42, 56, and 70 of terminal differentiation. All values were normalized to GAPDH and are presented as mean ± SEM (*n* = 3). Dunnett’s test was performed to evaluate the significance of groups compared with control (*p < 0.05). (TIF 384 kb)

## Abbreviations

Aβ_1–40_: Amyloid-β_1–40_
Aβ_1–42_: Amyloid-β_1–42_
AD: Alzheimer’s disease
AD pTAU: Alzheimer’s disease hyperphosphorylated TAU
APP: Amyloid precursor protein
APP-CTF: Amyloid precursor protein carboxy-terminal fragment
bFGF: Basic fibroblast growth factor
BSA: Bovine serum albumin
CHAT: Choline acetyltransferase
CLDN11: Claudin 11
DLG4: Discs large MAGUK scaffold protein 4
ELISA: Enzyme-linked immunosorbent assay
ER: Endoplasmic reticulum
fAD: Early-onset familial Alzheimer’s disease
FTD: Frontotemporal dementia
GAD1: Glutamic acid decarboxylase 1
GAD2: Glutamic acid decarboxylase 2
GAPDH: Glyceraldehyde 3-phosphate dehydrogenase
gDNA: Genomic DNA
GFAP: Glial fibrillary acidic protein
GRIN1: Glutamate ionotropic receptor *N*-methyl-d-aspartate type subunit 1
GRN: Granulin precursor
GSK3B: Glycogen synthase kinase 3β
ICC: Immunocytochemistry
IgG: Immunoglobulin G
iPSC: Induced pluripotent stem cell
LOAD: Late-onset Alzheimer’s disease
LTD: Long-term depression
LTP: Long-term potentiation
MAP2: Microtubule-associated protein 2
MAPT: Microtubule-associated protein TAU
NEAA: Nonessential amino acids
NES: Nestin
NF200: Neurofilament, heavy polypeptide 200 kDa
NFT: Neurofibrillary tangle
NIM: Neural induction medium
NMDA: *N*-methyl-d-aspartate
NMM: Neural maintenance medium
NPC: Neural progenitor cell
PAX6: Paired box 6
PFA: Paraformaldehyde
PHF: Paired helical filament
POL/L: Poly-l-ornithine/laminin
PSEN1: Presenilin 1
PSEN2: Presenilin 2
RBFOX3: RNA binding protein, fox-1 homolog 3
RT: Room temperature
sAD: Sporadic Alzheimer’s disease
SLC6A4: Sodium-dependent serotonin transporter
TBST: Tris-buffered saline with Tween 20
TD: Terminal differentiation
TH: Tyrosine hydroxylase
TUBB3: Tubulin β 3 class III
VACHT: Vesicular acetylcholine transporter
VGLUT1/2: Vesicular glutamate transporter 1/2
