# Supplementary figures and images for: Neurons derived from sporadic Alzheimer’s disease iPSCs reveal elevated TAU hyperphosphorylation, increased amyloid levels, and GSK3B activation
Source: Alzheimers Res Ther. 2017 Dec 1;9:90. doi: 10.1186/s13195-017-0317-z (PMC5709977; doi:10.1186/s13195-017-0317-z)

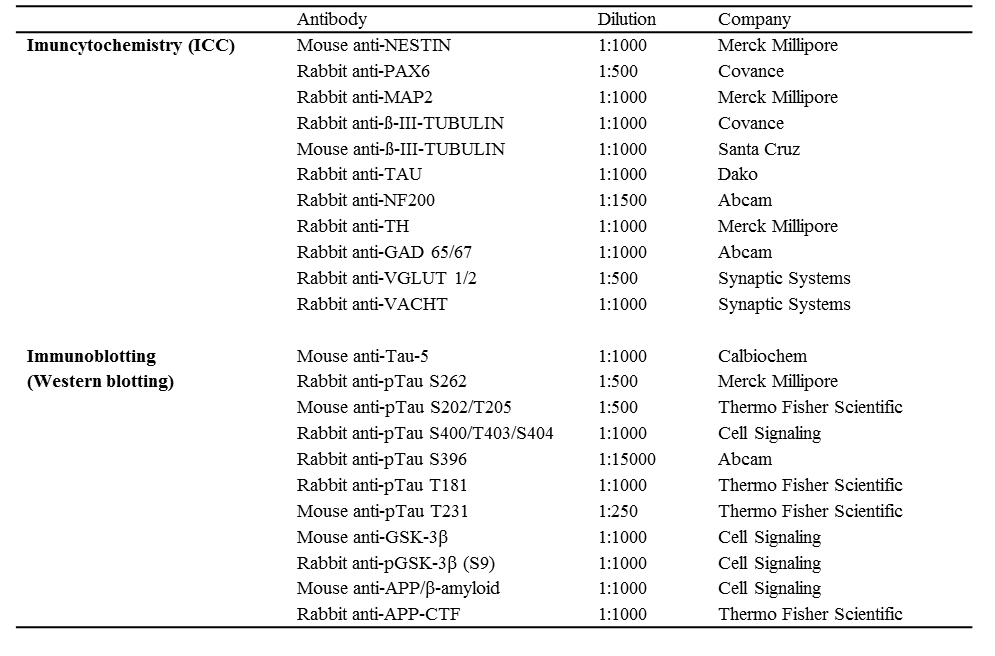

Supplement: Supplementary file 1 — Antibodies used in this study. (TIF 141 kb) [file 13195_2017_317_MOESM1_ESM.tif]

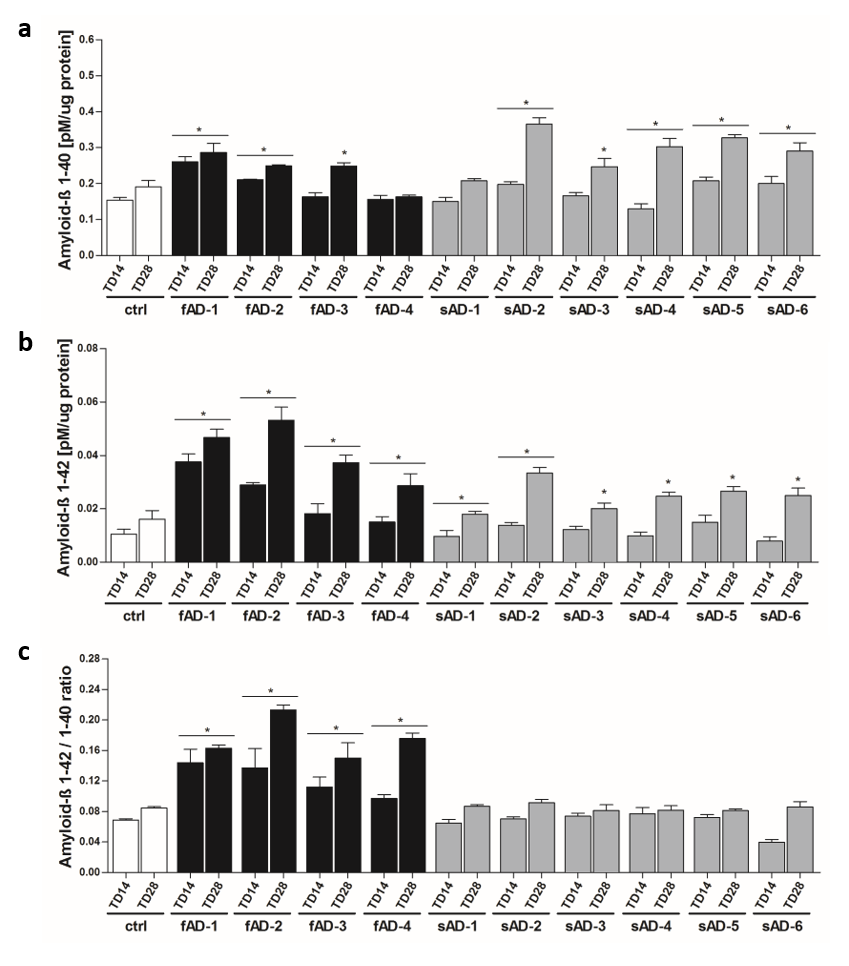

Supplement: Supplementary file 2 — Characterization of Aβ secretion in control and AD iPSC-derived neurons at TD14 and TD28. a The amount of secreted Aβ1–40 and (b) Aβ1–42 from control-, fAD- (fAD-1–fAD-4), and sAD- (sAD-1–sAD-6)-iPSC-derived neurons. c The ratio of Aβ1–42/Aβ1–40 from neurons derived from control and AD lines. Aβ1–40 and Aβ1–42 secreted from neural cells into the medium were measured at day 4 after the last medium change. The extracellular Aβ levels determined (in picomolar concentrations) were normalized to total protein content. Data represent mean ± SEM (n = 3). Because the detected levels of Aβ1–40 and Aβ1–42 were not significantly different between the four healthy individual-derived clones (ctrl-1, ctrl-2, ctrl-3, ctrl-4), the average value (±SEM) as a control value was used in all graphs. Dunnett’s test was performed to evaluate the significance of groups compared with control (*p < 0.05). (TIF 396 kb) [file 13195_2017_317_MOESM2_ESM.tif]

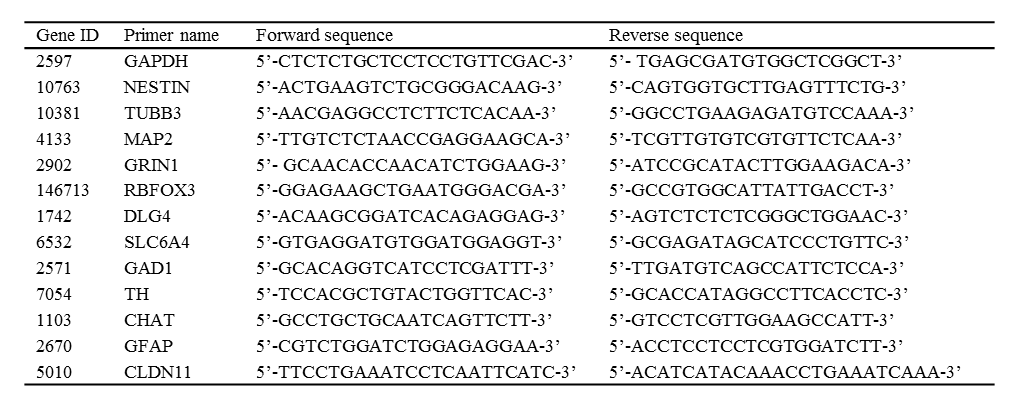

Supplement: Supplementary file 3 — PCR primers used in this study. (TIF 109 kb) [file 13195_2017_317_MOESM3_ESM.tif]

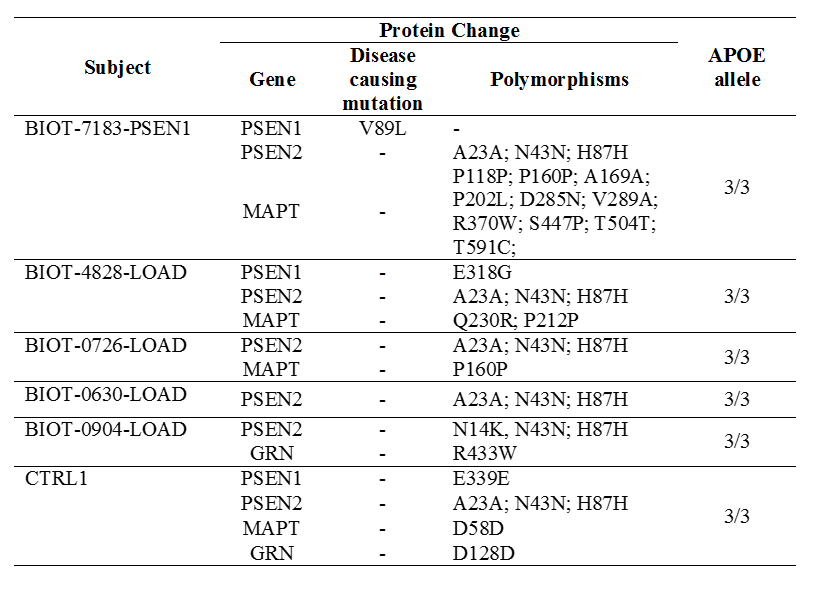

Supplement: Supplementary file 4 — Polymorphism of AD- and FTD-related genes and APOE status of cell lines used in this study. Footnote: Cell lines not detailed in the table were not permitted to be involved in whole exome sequencing; therefore, only clinical mutation identification was available. (TIF 130 kb) [file 13195_2017_317_MOESM4_ESM.tif]

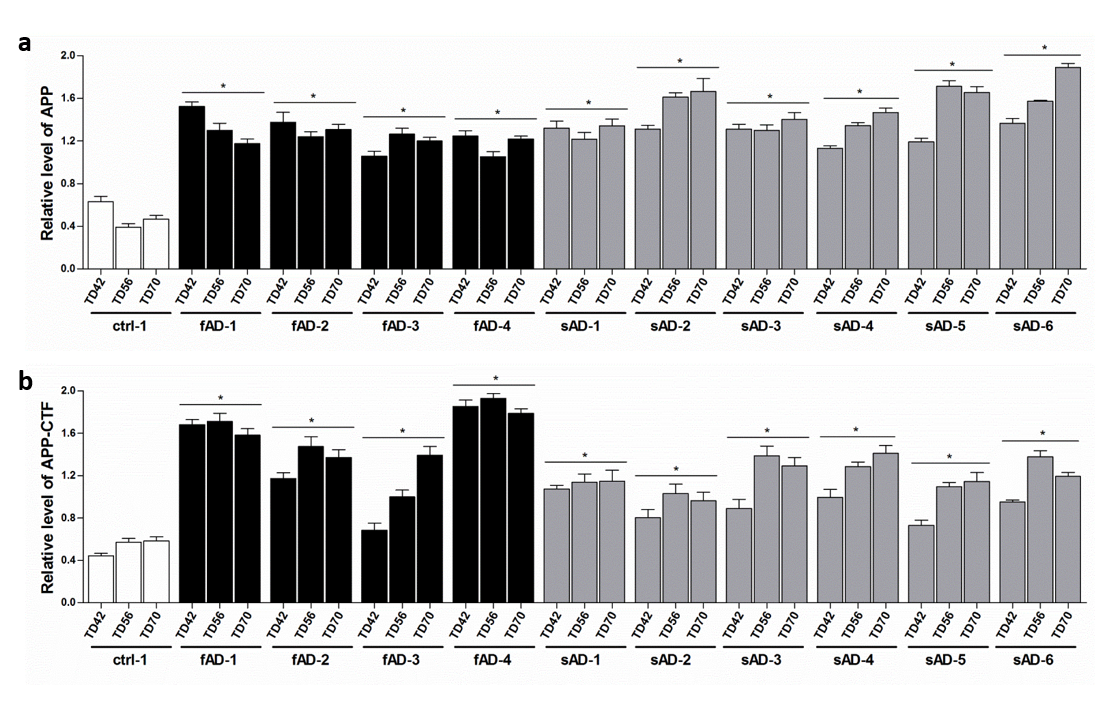

Supplement: Supplementary file 5 — Characterization of APP and APP-CTF expression during neuronal differentiation. Densitometric analysis of (a) APP and (b) APP-CTF expression in control neurons (ctrl-1), fAD-derived neurons (fAD-1–fAD-4), and sAD-derived neurons (sAD-1–sAD-6) at TD42, TD56, and TD70. Quantification of APP and APP-CTF signals was normalized to GAPDH. Data are presented as mean ± SEM (n = 3). Dunnett’s test was performed to evaluate the significance of groups compared with control (*p < 0.05). (TIF 1295 kb) [file 13195_2017_317_MOESM5_ESM.tif]

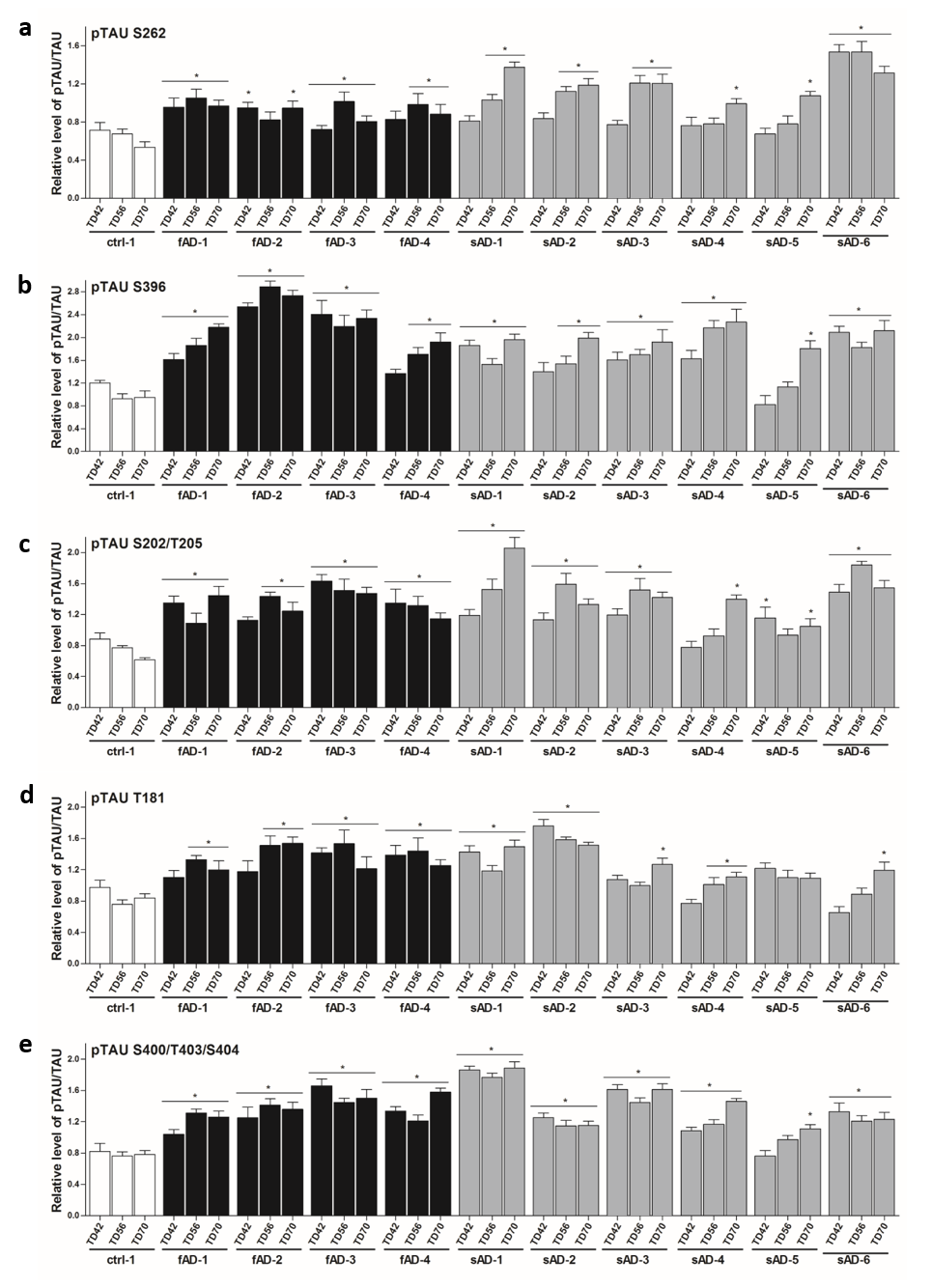

Supplement: Supplementary file 6 — Western blot analysis of total TAU and pTAU protein. a–e Densitometric analysis of TAU phosphorylated at different epitopes: S262, S396, S202/T205, T181, and S400/T403/S404. All samples were analyzed at days 42, 56, and 70 of terminal differentiation. The amount of pTAU relative to total TAU levels in the lysates was measured. GAPDH as the loading control was used to normalize the data. All values are the mean ± SEM (n = 3). Dunnett’s test was performed to evaluate the significance of groups compared with control (*p < 0.05). (TIF 804 kb) [file 13195_2017_317_MOESM6_ESM.tif]

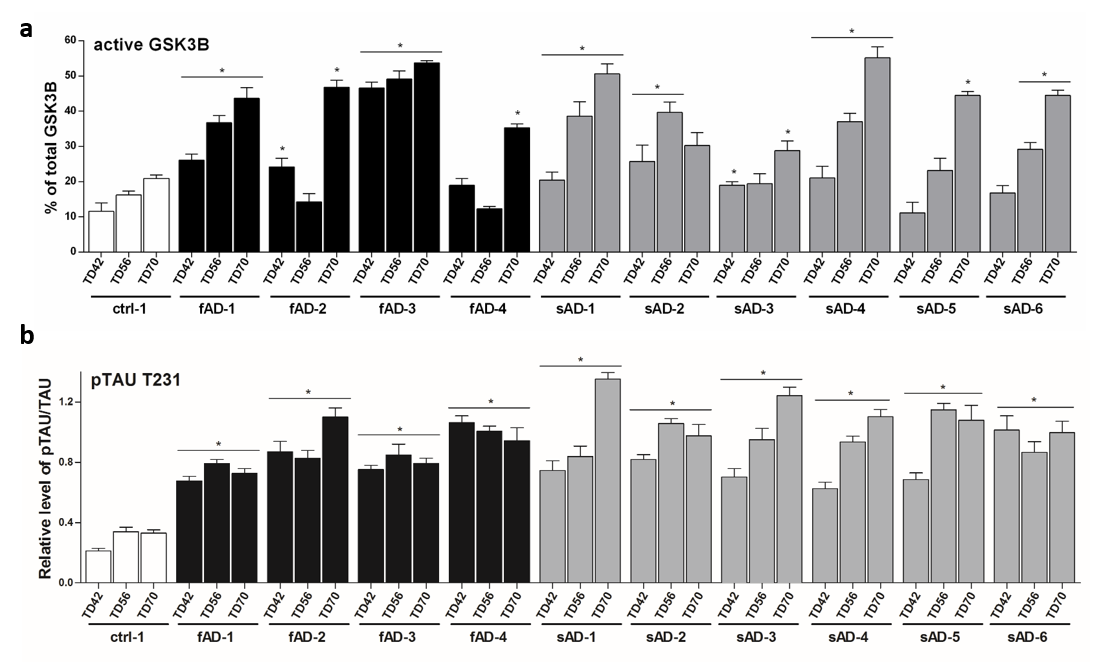

Supplement: Supplementary file 7 — Analysis of GSK3B and active form of GSK3B in neuronal culture. a Quantification of active GSK3B form in control neurons (ctrl-1), fAD neurons (fAD-1–fAD-4), and sAD neurons (sAD-1–sAD-6) at days 42, 56, and 70 of terminal differentiation was presented as a percentage of nonphosphorylated GSK3B at Ser9 (inactive form of the kinase [102]). b Densitometric analysis of TAU phosphorylated at T231 epitope at days 42, 56, and 70 of terminal differentiation. All values were normalized to GAPDH and are presented as mean ± SEM (n = 3). Dunnett’s test was performed to evaluate the significance of groups compared with control (*p < 0.05). (TIF 384 kb) [file 13195_2017_317_MOESM7_ESM.tif]
